# Dairy Buffalo Behavior: Calving, Imprinting and Allosuckling

**DOI:** 10.3390/ani12212899

**Published:** 2022-10-22

**Authors:** Daniel Mota-Rojas, Andrea Bragaglio, Ada Braghieri, Fabio Napolitano, Adriana Domínguez-Oliva, Patricia Mora-Medina, Adolfo Álvarez-Macías, Giuseppe De Rosa, Corrado Pacelli, Nancy José, Vittoria Lucia Barile

**Affiliations:** 1 Neurophysiology, Behavior and Animal Welfare Assessment, DPAA, Universidad Autónoma Metropolitana (UAM), Mexico City 04960, Mexico; 2 Consiglio per la Ricerca in Agricoltura e l’Analisi Dell’Economia Agraria (CREA), Research Centre for Engineering and Food Processing, Via Milano 43, 24047 Treviglio, Italy; 3 Scuola di Scienze Agrarie, Forestali, Alimentari ed Ambientali, Università degli Studi della Basilicata, 85100 Potenza, Italy; 4 Department of Livestock Sciences, Universidad Nacional Autónoma de México (UNAM), FESC, Mexico City 04510, Mexico; 5 Department of Agricultural Sciences, University of Naples Federico II, 80055 Portici, Italy; 6 Research Centre for Animal Production and Aquaculture, Consiglio per la Ricerca in Agricoltura e l’Analisi dell’Economia Agraria (CREA) (CREA), Via Salaria 31, 00015 Monterotondo, Italy

**Keywords:** maternal behavior, water buffalo, offspring, imprinting, nursing, allosuckling

## Abstract

**Simple Summary:**

Behavior during parturition not only allows us to understand the ethology of a species but can also be used as an early indicator of parturition. Although there are several studies on ruminants such as cattle, sheep, or goats, in the case of water buffalo, the study of their behavior and the initiation of maternal care is an area that requires further study. Therefore, this review aims to analyze the existing scientific evidence regarding maternal behavior in water buffalo during calving. It will address the mechanisms of imprinting, maternal care, and allosuckling strategies that may influence the survival and health of calves.

**Abstract:**

Maternal behavior, in water buffalo and other ruminants, is a set of patterns of a determined species, including calving, imprinting, and suckling. This behavior is mainly triggered by hormone concentration changes and their interactions with their respective receptors in the brain, particularly oxytocin. These chemical signals also influence mother–young bonding, a critical process for neonatal survival that develops during the first postpartum hours. Currently, dairy buffalo behavior during parturition has rarely been studied. For this reason, this review aims to analyze the existing scientific evidence regarding maternal behavior in water buffalo during calving. It will address the mechanisms of imprinting, maternal care, and allosuckling strategies that may influence the survival and health of calves.

## 1. Introduction

Water buffalo (*Bubalus bubalis*) are a species domesticated as a draught animal [1] and dual-purpose animal for meat and milk production [2]. In 2020, the global population of water buffalo was around 202 million, concentrated in Asia (97%), with 69% in India [1]. In particular, buffalo dairy farms are gaining importance due to the properties of buffalo milk, such as its high content of fat and proteins [3]. Since the success of dairy farms depends on parturition, lactation, and the welfare of both the dam and the calf [4], calving behavior in water buffalo is a research area that could help researchers understand this process, although it has rarely been studied in the species [5].

Currently, most of the available information regarding behavioral aspects during parturition can be deduced from other ruminant species, such as cattle, sheep, or goats [6,7]. Studies focusing on buffaloes describe behavioral patterns such as licking the newborn, water and feed-related activities, abdominal straining, and tail movements as some of the most common behaviors [8,9]. However, calving presentation depends on the parturition stage and the external aspects [10].

Recognizing a normal behavioral repertoire during calving can also help us to identify difficulties at birth for the purpose of preventive intervention in the case of dystocia [11,12], particularly in those cases that require surgical or obstetrical assistance to preserve the health of the mother and the newborn [12,13]. When mentioning calving difficulty, Srinivas et al. [14] mentioned that the majority of dystocia cases are due to maternal causes (59.16%), such as uterine torsion (83.33%), while fetal causes (40.84%) are due to the abnormal presentation of the fetus.

In addition to dystocia, stillbirth can also occur in buffaloes, a phenomenon where the calf dies within the first 48 h after parturition [15]. According to Salem et al. [16], the rate of stillbirths in Egyptian buffaloes increases due to factors such as the gestation length, birth weight, or parity. In this regard, in the same breed of buffaloes, the percentage of stillbirth in primiparous and multiparous females was 12.4% and 9.2%, respectively, and the backcrossing of Italian and Egyptian animals could diminish the presentation of stillbirths [17]. Therefore, since the success of dairy farms includes the reproductive health of females [18], studying calving behavior is part of the welfare protocol, not only during parturition but also in the first days of the life of the newborn.

For instance, after parturition, during the first six hours [19,20], imprinting and development of the mother–young bond are essential for neonatal survival and maternal care [21]. The presence of the calf, its vocalizations, and the placental fluids impregnated in the newborn’s coat are the cues that both the dam and the newborn use to establish the link and initiate maternal care [22]. Since one of the objectives of the selective care of ruminants is to provide the offspring with alternatives to decrease the mortality rate, calculated as 15.89% [23], 19.5% [24], and as high as 84% during the first month of the calf’s life [25], bonding is also related to early standing (preventing predator attacks or heat loss) [26], searching for the udder for prompt colostrum intake [27], and passive immunity transfer through feeding [28,29]. Moreover, although imprinting represents “exclusive maternal care for a biological calf”, in water buffalo, allosuckling and allonursing, known as the feeding and care of non-filial offspring, are regular practices observed in dairy farms [30]. Allonursing, although considered as a common event in other wild and domestic ruminants [31,32], has not been reported in numerous cases among water buffalo. The expression of these behaviors is still under study, but it could be due to maternal and offspring benefits that are more significant than the physiological cost they represent.

Thus, from an economic and welfare point of view [33,34,35], recognizing the buffalo’s behavior at calving can help us to promptly intervene when the expected repertoire is not present in order to prevent the consequences for the dam or the calf [36]. However, since most of the literature on calving relates to cattle or small ruminants, this review aims to analyze the existing scientific evidence regarding maternal behavior during calving in water buffaloes, as well as the possible calving complications that the species may present. Additionally, the mechanisms of imprinting and allosuckling will be discussed as elements that can affect the survival of water buffalo calves.

## 2. Calving Behavior

Maternal behavior is triggered by the onset of parturition, a period in which a set of particular behaviors are present due to hormonal stimuli and neuronal pathways [5]. It is essential to know which behaviors can be considered normal in the water buffalo during parturition, since this species may exhibit differences when compared to domestic cattle.

**Figure 1:**
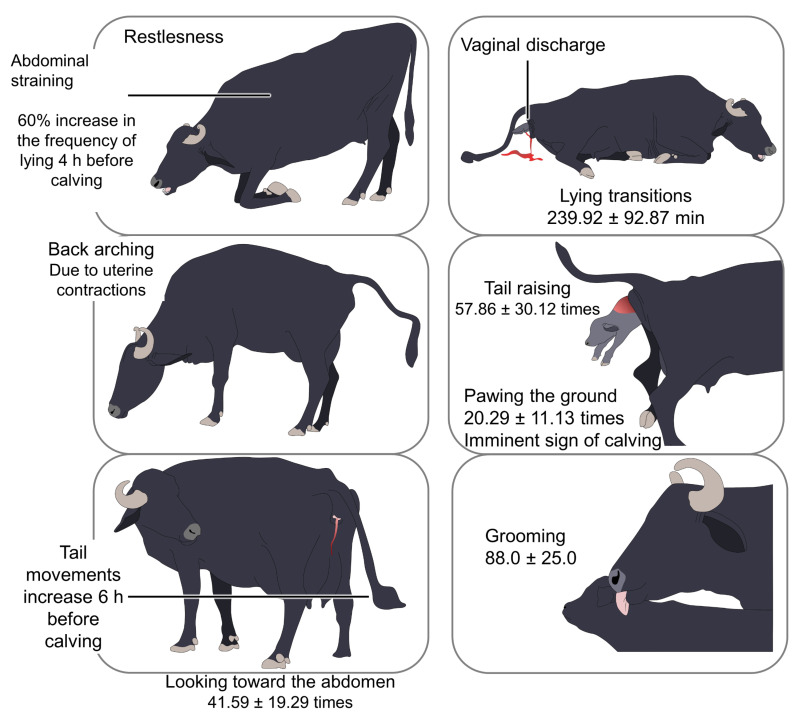
Main behavioral patterns during parturition in water buffalo. The exhibition of restlessness, back arching, and tail movements are considered good behavioral signs of the onset of parturition. Lying transitions, tail raising, or grooming are patterns observed during the last parturition stage, and their frequency or time of duration (assessed for 11 h pre-calving and expressed as number of times or min in the pictures) increases during calving.

According to Tulloc [8] and Das et al. [9], the most notable changes in the appearance of the buffalo occur two days before calving. Roberts [37] and Singh et al. [38] describe the licking of the newborn, interest in water and feed, abdominal straining, and tail movements as some of the most common behaviors (Figure 1). Days before the onset of the calving date, the spine and tail are highly flexible [8,9]. For these reason, Górriz-Martín et al. [39] highlight tail movements as an effective parameter to predict calving in cows.

The exhibition of these changes depends on the parturition phase. As stated by Deka et al. [40], during the first phase, restlessness, reduced interest in feed and water, lying transitions, tail raising, abdominal straining, back arching, vaginal discharge, and frequent urination predominate. The animal’s habit of looking towards its flanks, pawing the ground, and reduced feed intake are also present during this stage [8,9], and are associated with the onset of pain [41].

Water buffalo are a gregarious species with large groups; however, a buffalo cow isolates itself from the herd in order to look for a site that it considers protected [42]. This behavior, similar to cattle, helps to insure the survival of this species [30] and to guarantee two events after calving: imprinting and lactation [43]. Therefore, all these events can be considered as indicators that can be used to recognize the expected course of calving.

During the second phase, frequent urination is observed due to vaginal wall relaxation and bladder hypermotility [44], as well as the release of the placental fluids, after which the feet and/or head of the calf appear. Finally, in the third stage, the licking of the newborn, interest in food and water, and occasional abdominal straining are observed [40].

Lanzoni et al. [45] described the behavior of 20 Mediterranean Italian buffaloes monitored by video. They found that there was a 60% increase in the frequency of lying transitions from the control to the prepartum period. The walking frequency increased significantly until the prepartum period, which was most noticeable in the last eight hours before the parturition. In addition, the frequency of tail flicks or kicks against the ground and head-to-abdomen movements increased during the third, fourth, and sixth hours before calving. With these observations, it can be seen that the change in activity may be an early indicator of parturition in this species. In the domestic bovine, there is an 80% increase the standing periods among females housed indoors [46], and in this species, too, the change in activity may be an early indicator of parturition [47].

The behaviors described above may be due to pain perception [43]. Martínez-Burnes et al. [26] explain that during childbirth, inflammation of the myometrium and the release of proinflammatory substances help to maintain violent contractions for the expulsion of the product. However, the transmission of nociceptive stimuli through type C and type A fibers also occurs, resulting from the distension of the lower uterine segment and cervical dilatation [48,49]. Thus, the change in the activity level of the animals is also indicative of pain [50], and it may be used to assess the level of stress that cows and calves undergo during calving [36,51].

Currently, behavior during parturition in Holstein heifers and cows can be automatically evaluated using accelerometer-based devices placed on the tail of the animals before the onset of parturition [39]. In water buffalo, there are no reports on the use of these types of technologies to aid in the recognition of postural and activity changes, although this could aid dairy buffalo farms in predicting calving dates.

## 3. Calving Complication Behavior

### 3.1. Maternal Causes

As mentioned by Purohit et al. [11], calving complications, or dystocia, describe an event where the normal phases of parturition involve difficulties or are lengthened (more than 12 h), causing consequences for the mother and the newborn [52]. Compared to cattle (*Bos taurus*, *Bos indicus*), in water buffaloes, calving complications are reported less frequently (65.62% vs. 40.17%) due to the wide pelvic canal of the animals [11,53]. However, uterine torsion is the main cause of dystocia during the last stage of pregnancy [54], with rates of frequency of 53.57% [11] and as high as 83.33% [14].

**Figure 2:**
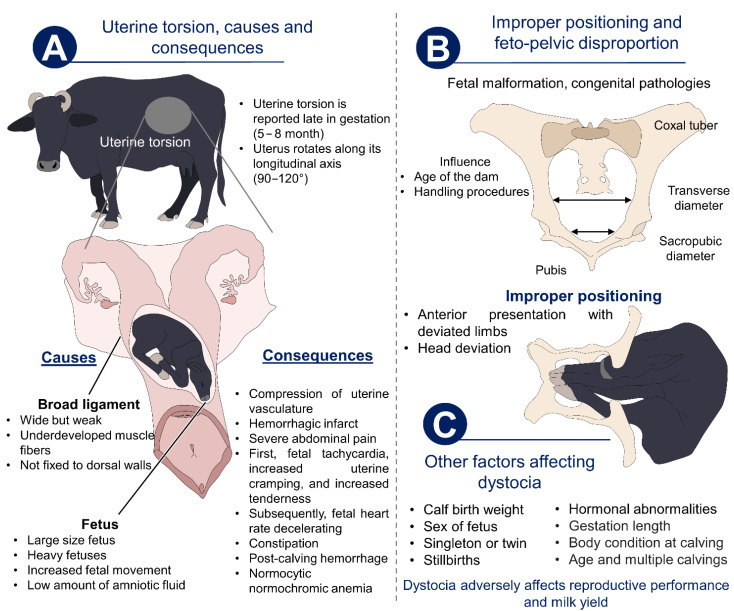
Maternal and fetal causes of dystocia in buffaloes. (**A**) Uterine torsion as the main complication during calving due to anatomical aspects such as a weak and underdeveloped broad ligament, as well as a large fetus, low amounts of amniotic fluid, and fetal movements. (**B**) Fetal–pelvis disproportions and improper positioning of the fetus are causes of dystocia that need to be treated or recognized early to avoid health consequences. (**C**) Other factors.

Uterine torsion causes the constriction of uterine blood vessels, an acute inflammatory process, and severe abdominal pain due to the stretching of the broad ligament [55] (Figure 2). Some of the behaviors that can be observed in dams are restlessness [56], kicking the abdomen with the hindlegs [55], pawing the ground, and back arching due to abdominal pain [57]. The lack of fetal membrane rupture is another indicator that could be considered as dystocia [57].

Prepartum vaginal and rectal prolapse have been also reported in buffaloes [58], with a prevalence of 43% [59], while placental retention represents between 4 and 18% cases of dystocia [60]. Although the survival rate of dams after treatment to correct maternal complications of calving is around 85.71% and 50%, the mortality rate of the dams tends to increase after 72 h following the first clinical sign of dystocia, resulting in consequences such as stress, peritonitis, toxemia, septicemia, dehydration, shock [14], and a reported 70% newborn mortality rate due to uterine torsion [61].

### 3.2. Fetal Complications

Fetal causes of buffalo dystocia represent 40.84% of cases [14]. Maternal–fetal disproportions [62] and the maldisposition of the fetus during calving [58] are common causes of dystocia-affected births in buffaloes. Considering disproportion, it has been reported that oversized fetuses are the cause of approximately 22.4% of dystocia cases [62], and this can be caused by anatomical abnormalities such as congenital deformities, ascites, and hydrocephalus, among others [14].

Regarding the presentation of the fetus during calving, animals in an anterior position (86.67%) and the deviation of the limbs (57.8%) and head (42.2%) are the widely most reported problems in buffaloes [14]. Maldisposition can cause stillbirth [63] and physiological alterations in the newborn such as hypothermia, hypercapnia, and the rupture of the umbilical cord, compromising the blood flow to the neonatal brain [58].

The consequences observed in both the dam and the calf constitute not only welfare and health issues for the animals but also an economic and reproductive barrier to the success of buffalo dairy farms. Therefore, monitoring the normal course of calving and identifying alterations during the process could help us to implement early measures and treatments. Figure 2 shows the maternal and fetal causes of dystocia in buffaloes.

## 4. Imprinting Mechanisms

Imprinting is known as the establishment of the selective bond during the first postpartum hours (up to six hours) [19,20]. It is a learning and endocrine process that activates neurological mechanisms to encourage maternal care [64]. In gregarious species such as the water buffalo, imprinting is essential for guaranteeing offspring survival [65], and in dairy cattle, authors such as Hudson and Mullord [66] stated that 5 min of contact with the newborn after parturition is enough to form the maternal selective bond. However, the separation of both for up to 5 h causes maternal unresponsiveness to the calf in 50% of the animals. Mora-Medina et al. [21] stated that the interruption of mother–young bonding interferes with calf learning and may hamper energy resource optimization.

This process requires both factors: the dam’s response and the learning ability of the neonate [22]. According to Orihuela et al. [67], dam’s response depends on a hormonal cascade in response to auditory, olfactory, tactile, and visual stimuli [21,68,69]. As a result, behavioral changes can be observed in the dam and the neonate [49,70].

There are scarce available data regarding water buffalo. Most of the research on the neuroendocrine response affecting maternal behavior has been developed by Poindron in the case of lambs and ewes [6] and by Numan and other authors in the case of rats [7]. The main hormones involved in maternal postpartum care are estradiol, progesterone, glucocorticoids, prolactin, oxytocin, and opiates (β-endorphin, metenkephalin, dynorphins) [20]. Neurotransmitters such as glutamate gamma-aminobutyric acid (GABA), norepinephrine (NE), epinephrine, dopamine acetylcholine (ACh), and nitric oxide (NO) are also related to maternal performance in ruminants [71]. The increase in placental estradiol in the prepartum period increases oxytocin and estrogen receptors (ER) in the medial preoptic area (MPOA), uterine endometrium, and mammary gland, while opiates have anxiolytic properties that promote OT activity. Additionally, OT favors maternal behavior, uterine contractility, lactation, and milk ejection [20]. For example, in cattle, salivary oxytocin concentrations during parturition were higher (1105 pg/mL) than those registered seven days after postpartum (7.1 pg/mL) [72], and this is related to vaginocervical stimulation [73] and the short sensitive period in which ruminants such as cattle and buffaloes develop maternal preference [74].

During the sensitive period, high estrogen concentrations and low progesterone are considered to activate oxytocin secretion in sheep and to increase the amount of estrogen receptors (ER-α) in the medial preoptic area [5,71,75]. This interaction is critical, since oxytocin is responsible for initiating maternal behavior, while estrogens maintain the above-mentioned behaviors [76]. In relation to this, although there are no reports on water buffalo, in other ruminants high estrogen levels and a low cortisol concentration are associated with an enhanced maternal performance [77].

Calf recognition requires the activation of cerebral structures such as the amygdala, hippocampus, and anterior cingulate, regions with large amounts of oxytocin receptors (OTR) [5]. In particular, the medial preoptic area is a critical region for developing maternal behavior [78], and its integrity is necessary for the successful performance of the dam [79]. Other regions, such as the nucleus accumbens, ventral tegmental area, lateral septum, the bed nucleus of the stria terminalis, and olfactory bulb, also participate in the endocrine cascade in order to develop certain behaviors [75].

The neuronal plasticity of the amygdala, hippocampus, and cerebral cortex during the first postpartum hours is the basis for the development of the mother–young bond [70], forming a neuronal connection through the axons and dendrites that can be life-long among the animals [80,81]. Therefore, an intervention in the sensitive period with a negative impact on imprinting could promote the rejection of the offspring [69], affecting its survival or the presentation of pathological behavior [82].

This process starts before parturition. In cattle, vaginocervical stimulation plays a key role in initiating maternal behavior [5]. The fetus’ expulsion stimulates sympathetic fibers in the vaginocervical region [21], activating hypothalamic oxytocin secretion [83]. Cytologic studies have shown that in the postpartum period, oxytocin immunoreactive cells and oxytocinergic cells increase in the dorsal raphe [84]. In addition to oxytocin, estrogen and dopamine concentrations are known to participate in the bonding process in ruminants [19,85]. Dopaminergic fibers project towards the mesencephalon, ventral tegmental area, nucleus accumbens, amygdala, and olfactory bulb, and each structure participates in recognizing the stimulus depending on its nature [86].

### 4.1. Tactile Stimulation

**Figure 3:**
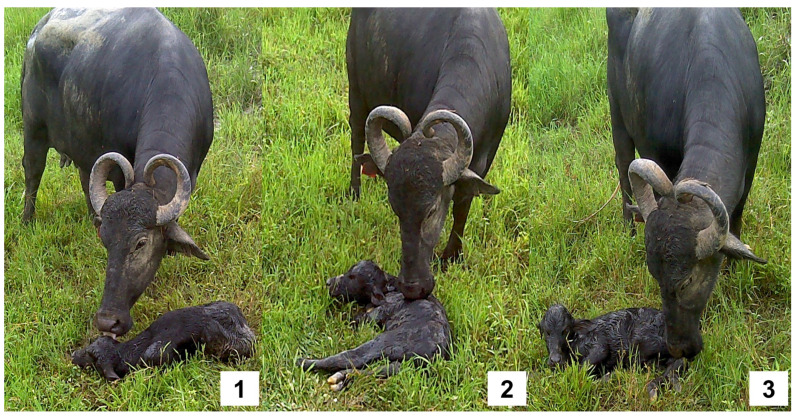
Sequential behavior of the licking of newborn water buffalo. (1) The beginning of grooming in the cranial region (nose, ears, mouth), moving towards the back (2) and ending in the anogenital region and limbs (3). This behavior can continue for up to 50 min. Although this is the regular pattern, the authors have detected that licking can start with the limbs and move in a cranial direction.

Tactile cues, such as licking or grooming, represent a reciprocal recognition of the to form a bond between the dam and calf, but they also help to obtain physiological benefits such as the encouragement of standing, prevention of heat loss [40,48,87], ingestion of amniotic membranes, cleaning of the nasal passages, stimulation of respiration, improvement of the muscular tone, and formation of the mother–young bond through odor cues transmitted through licking [88]. After calving, the dam engages in the active licking of the offspring, starting with the head, nose, mouth, and, sometimes, the eyes and continuing towards the back, rump, perineal region, and finally towards the feet (Figure 3) [8]. Through this behavior, the pheromones present in the saliva of the dam and prolactin secretion establish offspring recognition [89].

Genital stimulation also influences the attraction to placental fluids by the mediation of the olfactory function through oxytocin intracerebroventricular concentrations and the activation of norepinephrine neurons in the olfactory bulb, promoting licking [6]. Additionally, the presence of prolactin and placental lactogens promotes bonding. Deka et al. [40] reported that licking is present in the third stage of parturition, with a frequency of 100% among the 30 buffalo cows studied, a behavior (or a form of care) associated with decreased anxiety in the calf due to the influences of oxytocin and vasopressin, which have an impact on the response and brood abilities [90,91]. The role of oxytocin has been observed in studies using epidural anesthesia to block oxytocin release. In heifers exposed to this treatment, licking behavior was significantly reduced or eliminated (*p* < 0.05) due to the pelvic sensory deficits that impair oxytocin release. An important finding was that sensory stimulation was not enough in the case of nulliparous heifers to induce maternal behaviors, contrary to findings on ewes [92]

On the other hand, Dubey et al. [48], analyzing the postpartum behavioral activities of 25 Surti buffaloes, found that all the females spent more time sniffing and licking both the calf’s body and the perineal area. This stimulates respiratory and circulatory activity, ensures the elimination of urine and feces [93,94], and helps to avoid heat loss and hypothermia processes by eliminating amniotic fluids [95,96].

### 4.2. Olfactory Stimulation

Licking also promotes olfactory mother–young bonding [97,98]. This communication pathway is considered the most selective in the mother–young bond [99] because dams use their sense of smell within a close range to identify the newborn, facilitate lactation, and prevent misdirected parental care [40,100]. In the vomeronasal organ, the release of glutamate and GABA in the mitral cells, resulting in the activation of excitatory or inhibitory synapses in the olfactory bulb [101], enables olfactory learning through amniotic fluids and the perception of the olfactory cue by receptors in the vomeronasal organ [102] and the olfactory bulb [98,102]. However, studies show that olfactory recognition is mediated by the main olfactory system, while the accessory system (including the vomeronasal organ) exhibits contradicting roles, since it appears to be less relevant in domestic species than wild ungulates [103].

In this regard, anosmic ewes in Booth’s [104] study were unable to recognize their lambs and permitted the suckling of non-filial offspring, contrary to individuals with functional vomeronasal organ. These signals are later transmitted to the piriform cortex, orbitofrontal cortex, hippocampus, septum, amygdala, and hypothalamus, generating the observed behaviors and recognition of the presence of the newborn and its odor, as well as the endocrine response, where nitric oxide is another neurotransmitter that participates in olfactory memory [79]. Dubey et al. [48] reported that Surti buffaloes, classified as very attentive, sniffed and licked the calf’s body for a longer time (*p* < 0.05). The mother–young interaction has negative/positive effects based on the blood biochemistry of calves in dairy cattle. As stated by Wenker et al. [105], animals who had full contact during the first seven weeks postpartum had more health-related issues, such as increased levels of hematocrit and hemoglobin and a greater daily weigh gain (39.9 ± 1.3 kg), while mothers from this group yielded milk with lower percentages of fat (3.51 ± 0.13%).

Likewise, in ewes, treatments such as olfactory bulbectomy, the resection of olfactory nerves, or the use of substances that affect the functionality of the olfactory epithelium impede maternal recognition, making ewes incapable of distinguishing between filial and non-filial young [106]. Prepartum anosmia also affects selective maternal care in species such as goats and sheep [107].

### 4.3. Visual Stimulation

Another way to achieve adequate imprinting is visualization, used to recognize the offspring at a certain distance [67]. In cattle, vision is considered a main sensory organ in offspring recognition, although in water buffalo there are no studies focusing on this way of imprinting. In *Bubalus bubalis*, Rodríguez-González et al. [108] stated that visual recognition is achieved through optic nerve stimuli, which have connections with the occipital lobe, the lateral geniculate nucleus, and the temporal cortex. Together with tactile, olfactory, and auditory stimuli, oxytocin, a neurotransmitter involved in maternal behavior, is secreted [21,67,108].

### 4.4. Auditory Stimulation

Auditory communication, similar to visual cues, aims to facilitate long-distance and mutual recognition [109]. The vocalizations emitted by the calves in the first stage of their lives are perceived by the auditory cortex and the paraventricular nucleus [67,108,109,110]. In some studies, estradiol enhances the response of dams to the vocalizations of the newborn and, consequently, prolactin release also participates in this phase [20].

In ruminants, 2-day-old newborns can recognize acoustic signaling from the dam [111], and this period can be extended to include 3–5-week-old calves [112]. The significance of auditory cues is influenced by the age of the newborn. For instance, in lambs, visual recognition is more important than auditory imprinting [113]. In buffaloes, there are no studies regarding the influence of vocalization on maternal behavior; however, domestic calves have been shown to prefer and spend more time near speakers playing recordings of the mother’s vocalization than that of a non-filial cow [112].

Calf vocalization and maternal recognition of the vocal stimulus alters the behavioral response of the mothers. For example, in Holstein-Friesian cows, the separation from their calf disrupts the vocal patterns of the emitted sound, leading to open-mouthed calls, an increased number of calls, and shorter interval, between them, a behavior suggestive of a stress response in the cows [114]. Similarly, Padilla de la Torre et al. [115] found that cows did not only respond to the vocalization of their calf but also showed behaviors such as ear movement, looking, turning, or walking towards the sound, and this reaction was stronger in the dams of younger animals.

Therefore, in the imprinting period, the use of several sensory cues to form the mother–young bond in buffaloes is critical for reinforcing their communication. This trait is particularly advantageous for the neonate, since bonding implies selective maternal care and higher chances of survival.

## 5. Factors That Contribute to the Survival of the Offspring

The survival of the offspring is one of the main objectives of water buffalo production units, since it represents the reproductive success of the species and the consideration of factors that can alter it. Maternal behavior and care are designed to compensate for these factors and reduce neonatal mortality [43].

In general, the buffalo calf mortality rate has been estimated to be between 29.1% and 39.8% [116,117]. Other authors indicate an overall mortality of 42.11% [118] or 7.8% [119], depending on the housing, handling, feeding, and management of the animals [116]. Other factors responsible for perinatal loss include dystocia and subsequent hypoxia, as well as poor maternal and lactation behaviors, as they can contribute to starvation, low calf vitality, congenital abnormalities, infectious disease, and hypothermia [26,120].

**Figure 4:**
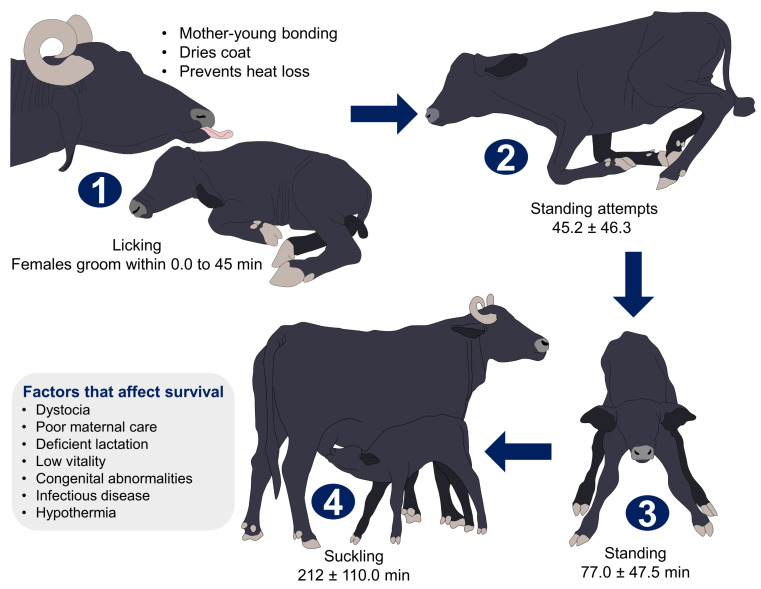
Factors affecting neonatal survival and characteristics of newborn calves during the first hours of life. The sequence of post-birth behaviors of the calf are numbered inside the figure, where the licking and grooming of the dam encourages the newborn to stand up and suckle. Under normal conditions, the offspring follows this pattern. However, factors such as dystocia, poor maternal care, and hypothermia, among others, can affect calf’s survival.

Firstly, the species-specific characteristics of newborn buffalo greatly contribute to their survival. As precocious animals, they can stand within the first 50 min of life, although their attempts can begin within the first 10 min [8,67]. The constant licking of the calf, starting with the nose and mouth and progressing to the anogenital region, is another behavior that encourages the calf to stand up [8], dries their coat, and participates in the oxytocinergic endocrine cascade of mother–young bonding [121]. In 30 primiparous Italian Mediterranean buffaloes, Lanzoni et al. [45] found that, in the first 6 h after parturition, the dams spent intervals of 7.7 ± 2.5 min grooming the calves, a behavior that gradually decreased (Figure 4) [45].

Standing prevents another main factor that can affect offspring survival: hypothermia [26]. Hypothermia presents as a response to the thermal extrauterine challenge, where a decrease in body temperature of up to 2 °C can be recorded [122]. However, buffalo calves are born with anatomophysiologic traits, such as skin thickness, a high melanin density, thick hair follicle arrangement [123], and poor sweat gland arrangement, which impede heat dissipation in hot climates [124].

Lezama García et al. [125] stated that the water buffalo, being a precocious species, can compensate for the decrease in temperature through their brown adipose tissue or by shivering [126]. This could be one of the reasons why the water buffalo have a certain resilience to the cold, as the anatomical characteristics prevent the thermal exchange of heat with the environment [30], resulting in a lower incidence of neonatal mortality, being around 0.51–1.61% [127,128,129]. Although these percentages are not exclusive to mortality due to hypothermia, Boro et al. [130] stated that factors such as the time of year influence the increase in mortality in neonate ruminants.

**Figure 5:**
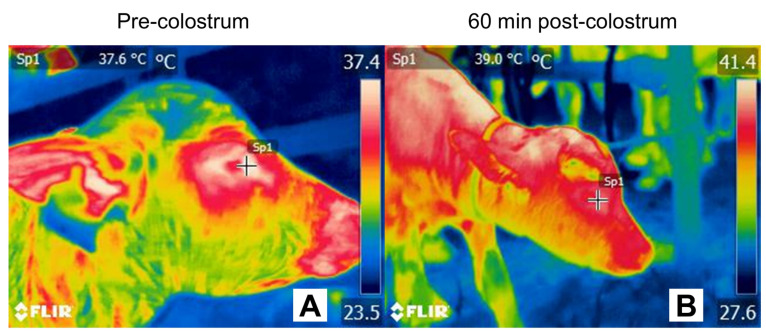
Effect of colostrum on thermoregulation in the newborn calf (*Bubalus bubalis*). (**A**) Lateral facial thermogram of a calf before receiving colostrum. The temperature assessed at the lacrimal caruncle shows an average value of 37.6 °C. In contrast (**B**), 60 min post-colostrum intake, the same thermal window has an average temperature of 39.0 °C. This represents a gain of 1.2 °C after feeding and rapid obtainment of available energy resources in colostrum. +Sp1: default focal point of the software. Thermal images obtained using a FLIR thermal camera.

The ability to stand up for colostrum consumption participates in the thermoregulation of buffaloes [125,131,132,133]. Colostrum intake is a crucial element for the survival of newborn ruminants and largely depends on adequate imprinting mechanisms immediately after calving [26]. Neonatal calves have low energy stores and need to obtain an adequate colostrum volume in the first 6–7 h [27]. Colostrum satisfies the metabolic needs of newborns and is also the main source of immunoglobulins, growth factors, peptides, enzymes, and hormones [134]. For this reason, in buffalo newborns, as in other species of ungulates, colostrum provides resources necessary to prevent infections during the first hours of life, given its role in passive immune transfer [28], and offers energy for thermoregulation [26] (Figure 5).

Suckling behavior in calves is influenced by the time when they suckle. Teat-seeking is the first behavior observed in ruminant neonates after birth, with an average of 173 min from the first standing to the first teat-seeking event [135]. Normally, in almost 67% of mother–young interactions, calves suckle on all four teats [120], and other behaviors, such as scratching, sniffing, and butting, are milk stimulation responses [136] associated with females with low milk flow rates [137], a trait that might influence alter the nutritional properties of colostrum.

Nagyová et al. [134] found that the total protein profile was significantly increased in calves, lambs, and kids at 24 h after birth, and they also identified a high concentration of hormones and growth factors [138]. This response is related to the quality and nutritional components of the colostrum. In the case of water buffalo, the colostrum has higher percentages of fat (range of 7.56–11.22), lactose, vitamins, and total solids (26.67%) compared to that of cows of the same age and time to calving [139,140]. Likewise, the suckling behavior and amount of colostrum uptake depends on the milk availability and production of the dam. In fact, calves of dams with a low milk flow tend to have more ineffective suckling bouts, and this can be altered by the teat composition [120]. Table 1 summarizes the colostrum composition during the first postpartum day in water buffaloes.

Delayed suckling hinders colostrum intake, leading to starvation and hypothermia [143]. Buffalo calves take an average of 212 ± 110 min to achieve their first suckling, a behavior that lasts for approximately 38 ± 22 min during the first 6 postnatal hours [45]. Likewise, Andriolo et al. [144], in a study on 29 Murrah-Mediterranean buffaloes, observed that calves spent an average of 11 min per day lactating. Moreover, calf–dam interaction can also compensate for hypothermia and prevent its physiologic consequences [67]. These results confirm that maternal care is a crucial factor affecting the survival of the offspring [21,131].

Since colostrum is associated with immune function in the neonate, the delay in colostrum intake may influence disease incidence. For example, in dairy buffalo farms, endo-parasite infestation (83.3%), diarrhea, and ectoparasites (each 81.3%) were the most frequent diseases in farms where colostrum was not provided immediately after birth (delay of about 7–8 h after birth), resulting in an average mortality rate of 79.51% [145]. Similarly, Ullah et al. [116] reported that common causes of mortality increase in buffalo calves are diarrhea and pneumonia as consequences of delayed colostrum feeding, causing worm infestation and gastrointestinal obstruction, subsequently causing the death of approximately 17.98% of calves [83]. Due to the importance of colostrum in the development and survival of the calf, in extreme cases, where the mother is not able to feed the newborn, feeding colostrum techniques could be implemented to provide the nutritional benefits. Techniques such as bottle feeding or tube feeding have shown a reduced rate of immunoglobulin transfer failure (19.3%) in comparison to animals fed by their mothers (61.4%) [146]. Likewise, between stomach tube feeding, nipple feeders, and natural suckling, in 15 newborn calves, the animals fed with stomach tubes had higher levels of serum IgG (15.74 ± 0.29 mg/mL) and a reduced morbidity rate (20%), without a difference in their height and length gain [29], showing that artificial feeding has benefits in calves.

Therefore, it is possible to declare that climatic and physiological factors contribute to the performance of the calf in order to guarantee its survival during suckling. Nonetheless, maternal behavior and care greatly contribute to the health of buffalo newborns.

## 6. Maternal Care of the Newborn and Allosuckling

Maternal care represents selective nursing behaviors exhibited towards the offspring [147], where suckling is the first attachment mechanism required for the dam to develop maternal behavior [5,148]. As indicated in a study by Dubey et al. [48], in Surti buffaloes, calves of dams with a low maternal behavior score (MBS) spend a longer time finding the teat (105.23 ± 8.60 vs. 102.87 ± 10.75) and have a higher number of standing attempts (34.24 ± 2.09 vs. 32.63 ± 3.52) compared with buffalo calves with a high-MBS dams, which could suggest that maternal behavior directly influences neonatal behavior [48].

Currently, the evidence suggests that oxytocin, progesterone, estrogen, prolactin, and testosterone levels during pregnancy regulate maternal behavior, contributing to the strengthening of affiliative responses [5,43,148,149]. The effects of these hormones on the medial preoptic area motivate maternal care and lactation [148,150,151]. Through visualization, olfactory, auditory, and tactile cues, and their transmission to the medial preoptic area, the effects on the paraventricular nucleus and the supraoptic nucleus cause oxytocin and vasoactive intestinal peptide release, promoting the contraction of myoepithelial cells around the ducts and alveoli to promote milk removal and prolactin release [152,153,154,155,156].

Given this effect, a study on Italian Mediterranean buffaloes found that dams performed grooming (licking + sniffing) to a greater extent during the first day of calving compared to the second day. In addition, the results showed a negative correlation between maternal rejection and the daily weight gain at 14 and 21 days [45].

On the other hand, interactions within the group contribute to the initiation of specific strategies, such as allonursing, a form of parental care widely exhibited by diverse ungulates with a synchronized social hierarchy and reproductive cycle, such as the water buffalo [157,158]. This behavior consists of the feeding of non-filial offspring [158] without the neglect of the cow’s own offspring when allowing allosuckling [144], and it does not occur unless the offspring calf solicits suckling from another buffalo cow and is willing to accept it [30,152]. Murphey et al. [159] demonstrated that in Murrah-Mediterranean buffaloes, the acceptance pattern of cows suckling non-filial calves was mostly observed in dams that did not reject their offspring and motivated non-filial calves to engage in communal suckling [159].

**Figure 6:**
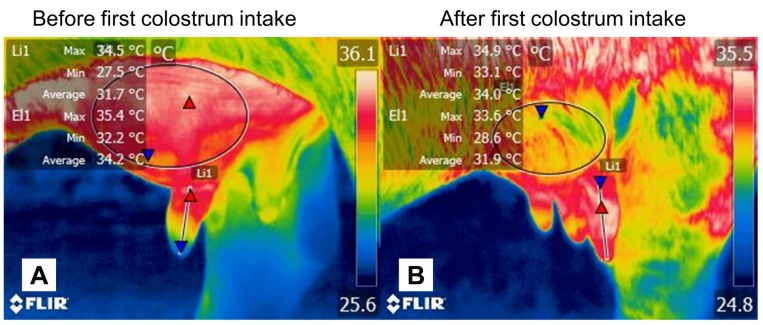
Microcirculatory changes in a water buffalo (*Bubalus bubalis*) udder before and after colostrum ejection. (**A**) Lateral region of the udder before the first colostrum intake. The average temperature of the teat (Li1) is 31.7 °C, a temperature that is lower than the average recorded in the body of the gland, at 34.2 °C. This could be due to the effect of estrogens and the congestion of the mammary gland (MG) before feeding the newborn. (**B**) After the first colostrum intake, a shift in the average temperatures is observed, where the teat has a 2 °C higher value, and the body of the MG has an average temperature 2.3 °C lower than the one observed in image (**A**). This effect can be attributed to the effect of colostrum ejection, where the calf stimulates the teat, suckles, and requires a larger supply from this zone. The maximum temperature is illustrated with a red triangle and the minimum with a blue one. Thermal images obtained using a FLIR thermal camera.

With respect to this novelty, it is believed that buffaloes that exhibit more than one suckling behavior are more stimulated in terms of milk production [160], which shows with parallels with the findings of Murphey et al. [152], who observed that calves with dams who produced less milk were more likely to request nursing from, and be nursed by, other buffaloes. Likewise, the authors indicated that this behavior could be influenced by the hunger level of calves, which could increase the intensity of solicitations due to milking, favoring suckling by inexperienced buffaloes. Additionally, allosuckling may represent an advantage for the dam, considering that estrogens participate in lactation physiology, causing venous congestion in the udder (*vena epigastrica cranialis superficialis* and *vena subcutaneous abdominalis*) before the newborn suckles (Figure 6) [161]. Therefore, in females with high milk production, feeding non-filial calves could prevent mastitis as a milk-dumping alternative [30].

However, factors such as gender have a decisive influence on social interactions during suckling, whereby dams of the Murrah, Jaffarabadi, and Mediterranean breed with bull calves tend to exhibit higher milk production and more frequent suckling compared to buffaloes with heifer calves. This could support the hypothesis regarding differential maternal investment according to the sex of the calf [160,162].

## 7. Perspectives

The behavioral response of buffaloes during calving is relevant for farmers’ efforts to understand the course of an eutocic process. However, as in the case of cattle, where research on this area is focused on the so-called “digital agriculture” or precision livestock farming (PLF) [163], based on the use of technologies such as accelerometers to determine patterns such as the location preference for parturition in Holstein heifers and cows [164], in water buffalo, this technology could be used for the same purpose. However, limited studies on the use of these devices to assess the activity, resting, eating, and rumination behaviors not associated with parturition have been conducted [165]. Likewise, while there are reports indicating that the ideal climatic temperature for reproduction in water buffaloes is 13–18 °C, with a relative humidity of 55–65% [134], from a behavioral perspective, it would be interesting to know whether environmental conditions affect the exhibition of certain calving behaviors or if maternal aspects of the dam influence the survival of the newborn [166]. The effects of the parity number and maternal experience are other aspects that remain unknown in buffalo farms, since few studies regarding parity have been conducted for this species [167]. Nava-Trujillo et al. [168] found that primiparous buffaloes had longer average lactation (310.05 to 268.05 days in the third parity) and average calving intervals (497.45 to 418.39 days), parameters that improved with parity. A similar result was observed in ewes with previous maternal experience, as individuals who had a higher amount of estrogen receptors in the paraventricular nucleus, medial preoptic area, and medial amygdala, markers associated with a higher performance [169]. However, there is no report on the associations of maternal performance in buffaloes, in contrast to information regarding cows, reporting that heifers licked their calves less frequently than cows and suckled their newborns for a shorter time, suggesting that cows are better dams [170].

When evaluating the imprinting process, the further evaluation of the endocrine components could expand our understanding of the mother–young interaction in this species [171]. For example, Hernandez et al. [172] determined that oxytocin levels were raised in goats as a response to nursing their kids, but no effect on the prolactin concentration suggestive of an association of maternal selectivity with this hormone was observed. Derar and Abdel-Rahman [173] also assessed the hormonal response in heifers and cows during eutocia and dystocia. The authors found that dystocia activated an adrenal response (elevated cortisol levels), affecting the physiological parameters of both the dam and the calf, aspects that could alter the imprinting process. Additionally, higher vagal activity has been reported in cow dams during the suckling of their offspring [174]. In buffalo, most of the available data are obtained from ruminants in general. Therefore, studies focusing on this species are necessary, although, considering the productivity of the animals, some authors state that acoustic, olfactory, and manual stimuli do not influence milk let-down [174].

On the other hand, despite the fact that allosuckling and allonursing have been observed in buffaloes [21,30,162], the roles of the different hypotheses regarding this altruistic behavior remain to be an under-researched area. Social benefits and misdirected parental care have been studied in other species, such as meerkats [175] and guanacos [176]. The immunological traits, rather than representing a benefit, as in the case of animals such as rodents [177], in water buffalo calves are related to disease predisposition [133]. Therefore, the influences of maternal and offspring characteristics need to be studied in-depth in buffaloes so as to understand the different maternal behaviors observed in the species [132].

## 8. Conclusions

The study of calving behavior in *Bubalus bubalis* represents a tool that can be used to improve our ethological knowledge of the species, but it can also be used to determine the normal course of parturition. However, in addition to its importance for diary buffalo farms, the present article also highlights some of the available literature regarding calving behavior. According to the parturition phase, behaviors such as restlessness, frequent lying down, tail raising, or back arching are the main postural changes. These are responses with a physiological and endocrinological basis, where parturition pain can trigger these changes. The recognition of these changes can also help us to identify when dystocia could occur due to maternal or fetal causes that might alter the behavioral response of the dam.

Together with the behavioral response, alterations in hormonal levels participate in the onset of parturition and play a key role during the imprinting process. Oxytocin, dopamine, and the action of several cerebral structures, such as the mesencephalon, nucleus accumbens, paraventricular nucleus, and hypothalamus, control maternal behavior. Through auditory, olfactory, visual, and tactile cues, both the dam and the newborn establish a bond that contributes to neonatal survival. Through dams’ recognition of their offspring, maternal care is exclusively directed towards the calf, starting with grooming to encourage standing and early colostrum intake, an essential element enabling ruminants to obtain readily available energy resources and passive immune transfer.

Although imprinting signifies an exclusive bond with the neonate, the occurrence of allosuckling and allonursing in water buffalo is an ongoing research field, representing processes through which the dam and the calf can obtain benefits, such as nutritional compensation when the female does not yield an adequate quantity of milk, or acts that improve the dams’ reproductive performance and maternal ability. It is essential that all these factors are considered in dairy buffalo farms, since they not only have consequences for the productivity of the animals but are also linked to their welfare.

## Figures and Tables

**Table 1:** Chemical and immune composition of colostrum during the first postpartum day in water buffalo.

	IgG (g/L)	IgA (g/L)	IgM (g/L)	Lactose (g/100 g)	Fat (g/100 g)	Protein (g/100 g)	References
Colostrum composition	33.20	3.22	3.00	4.3	10.4	5.9	[139,141,142]

Ig: immunoglobulin.

## Data Availability

Not applicable.
